# Nanotheranostic Applications for Detection and Targeting Neurodegenerative Diseases

**DOI:** 10.3389/fnins.2020.00305

**Published:** 2020-04-30

**Authors:** Ajay Kumar, Ravi Kumar Chaudhary, Rachita Singh, Satya P. Singh, Shao-Yu Wang, Zheng-Yu Hoe, Cheng-Tang Pan, Yow-Ling Shiue, Dong-Qing Wei, Aman Chandra Kaushik, Xiaofeng Dai

**Affiliations:** ^1^Institute of Biomedical Sciences, National Sun Yat-sen University, Kaohsiung, Taiwan; ^2^Department of Mechanical and Electro-Mechanical Engineering, National Sun Yat-sen University, Kaohsiung, Taiwan; ^3^Department of Biotechnology, Institute of Applied Medicines & Research, Ghaziabad, India; ^4^Department of Electrical and Electronics Engineering, IIMT Engineering College, Uttar Pradesh Technical University, Meerut, India; ^5^School of Computer Science & Engineering, Nanyang Technological University, Singapore, Singapore; ^6^Department of Physical Medicine and Rehabilitation, Kaohsiung Veterans General Hospital, Kaohsiung, Taiwan; ^7^Key Laboratory of Microbial Metabolism, School of Life Sciences and Biotechnology, Shanghai Jiao Tong University, Shanghai, China; ^8^Wuxi School of Medicine, Jiangnan University, Wuxi, China

**Keywords:** neurodegenerative disorders, nanotheranostics, Parkinson’s disease, Alzheimer’s disease, nanocomposite

## Abstract

Nanotechnology utilizes engineered materials and devices which function with biological systems at the molecular level and could transform the management of neurodegenerative diseases (NDs) by provoking, reacting to, and intermingling with target sites to stimulate physiological responses while minimizing side effects. Blood–brain barrier (BBB) protects the brain from harmful agents, and transporting drugs across the BBB is a major challenge for diagnosis, targeting, and treatment of NDs. The BBB provides severe limitations for diagnosis and treatment of Alzheimer’s disease (AD), Parkinson’s disease (PD), and various other neurological diseases. Conventional drug delivery systems generally fail to cross the BBB, thus are inefficient in treatment. Although gradual development through research is ensuring the progress of nanotheranostic approaches from animal to human modeling, aspects of translational applicability and safety are a key concern. This demands a deep understanding of the interaction of body systems with nanomaterials. There are various plant-based nanobioactive compounds which are reported to have applicability in the diagnosis and treatment of these NDs. This review article provides an overview of applications of nanotheranostics in AD and PD. The review also discusses nano-enabled drug delivery systems and their current and potential applications for the treatment of various NDs.

## Introduction

Technological developments across various fields have proven to be beneficial for the advancements of medical research. These developments have assisted in developing various novel and efficient techniques, machines, and methods which can cure different challenging conditions and disorders of the human body. In addition to these technological advancements, an increase in research in the field has also led to the development of various techniques which can help in combating different disorders (Bronzino, 2014). One such recent development is nanotheranostics, which has been defined by Lammers et al. (2010) as an approach which integrates target-specific diagnostics as well as therapeutics for complete recovery from the disease. These integrated practices are dependent on the use of different nanotechnology platforms, which are specifically designed for being able to target a given physical condition or disorder and hence can be used for both diagnostics and therapeutics purposes. One of the most common applications of this therapy or practice is in the treatment of neurodegenerative disorders, which are caused by different hereditary or sporadic conditions and mostly result in various dysfunctions of the nervous system of patients (Sharma, 2014). This review provides an overview of applications of nanotheranostics in neurodegenerative diseases (NDs) and recent advancements in nano-enabled drug delivery systems.

## Discussion and Analysis

### Neurodegeneration

Neurodegeneration, as explained by Williams (2002), is the basis of all neurodegenerative disorders like Parkinson’s disease (PD), Huntington’s disease, and Alzheimer’s disease (AD) and is primarily used to describe any condition which tends to affect the normal functioning of neurons in the brain. Williams (2002) explained that neurodegeneration combines two words, “neuro” and “degeneration,” and hence refers to the degeneration of neurons, which in turn affects memory and other cognitive abilities of the patients. There are several researches which focused on the analyses of these neurological disorders. While several people assume it to be incurable, latest findings have revealed that the use of new techniques and approaches like nanotheranostics can render better outcomes. In addition to genetic mutations, other factors which have been found to result in neurodegeneration among patients include the buildup of different toxic proteins in the brain and the formation of neurotoxic molecules that can be caused by the loss of mitochondrial functions (Ingelsson, 2016). Further discussions explain the symptoms and molecular mechanisms associated with some of the neurodegenerative disorders which will help to understand the effectiveness of various nanotheranostic approaches.

### Common Neurodegenerative Disorders

#### Alzheimer’s Disease

Alzheimer’s Disease is one of the most commonly observed neurodegenerative disorders across the world. According to Glenner and Wong (1984), it is one of the major causes of dementia among older patients, which further leads to the death of huge masses of people in developed countries across the globe. According to the data presented by Vos et al. (2015), more than 25 million people in the world are suffering from AD at present, among which the most prevalent countries being China, Western Europe, and Latin and North America. The total incidences of AD in the world are expected to rise to almost 42.3 million by the end of 2020 (Das et al., 2012). The key implication of the disorder which is witnessed in patients suffering from this disease is neurodegeneration, which may be initiated by protein deposition or synaptic injury and then results in neuronal loss, which tends to cause memory loss, cognitive disabilities, and behavioral changes in the patients (Garand et al., 2000).

Although there have been several studies that explain the molecular mechanisms associated with the disease, Sastre et al. (2006) argue that it continues to be unclear as to which of the mechanisms are most suitable and relevant in describing the occurrence of AD in people. One of the most commonly discussed molecular mechanisms used to explain the disorder is the hyperactivation of cyclin-dependent kinase-5 (CDK5), which is a signaling protein. As explained by Masters et al. (2006), CDK5 is one of the most important proteins which is responsible for neuronal development and usually gets activated by the generation of p35 or p39, which ultimately results in the formation of a specific complex, which tends to become a very important element of the overall neuroplasticity as observed in the pathogenesis of the disorder (Masters et al., 2006).

The molecular mechanism occurring during the interaction of CDK5 with p25 leads to over activation of CDK5 and also results into tau hyper phosphorylation, and these two occurrences collaboratively lead to the formation of neurofibrillary tangles (NFTs), which results in neural disorder (Dong et al., 2009). The cleavage of the activators has been found to occur because of the abnormal processing of amyloid precursor protein (APP) due to genetic disorders, which results in the changes in overall calcium signaling. The model suggests that the p25 activator is associated with the memory of people and hence over generation of the same in patients’ brains results in memory loss (Giese, 2014).

However, the recent discussions presented by Lloret et al. (2015) confirmed that there is actually a decline and not increase in the quantity of p25 in AD. While there have been several debates regarding the same, Lloret et al. (2015) explained that the reduction in overall quantity of p25 leads to higher levels of amyloid-induced synaptic toxicity, which eventually results in a reduction in calcium signaling, thus causing degeneration of the neurons. In addition to this, there is yet another model that describes the occurrence and molecular phenomenon and mechanism associated with the disease which is related to abnormal accumulation of amyloid-β (Aβ) and plaques containing the same, which finally results in toxic oligomers and neuronal disorders (Rajmohan and Reddy, 2017). Thus, these molecular changes and reactions have been found to be the main causes of AD and will further help in understanding the application of nanotheranostics for treating the same.

#### Parkinson’s Disease

The second most common neurodegenerative disorder is PD, which is a disorder of the central nervous system (CNS), thus leading to various problems in motor abilities and system of the patients. As explained by De Lau and Breteler (2006), the disorder leads to malfunctioning or even death of the different vital neurons in the brain, especially those present in the substantia nigra region. The death of these neurons leads to a decrease in the production of dopamine (DA), which is a key chemical and is used for instructing brain to carry out and control different activities and movements. Thus, a reduction in DA leads to impaired movement and affects other body functions (Mazzoni et al., 2012). Some of the key signs or symptoms of PD include stiffness of limbs, slow movement, tremor in hands and other joints, and difficulty in balance and coordination of different body parts and movements (Jankovic, 2008). Although the exact cause of PD has not been established yet, studies have explained the molecular mechanisms underlying the same that help in explaining the occurrence of the disease.

One of the key molecular mechanisms that is found to be associated with the onset of PD is the development of filamentous structures of the major protein constituent of the brain, i.e., alpha-synuclein (α-synuclein), which results into the development of toxic materials (Burré et al., 2010). As explained by Stefanis (2012), the protein α-synuclein plays an extremely pivotal and significant role in the recycling and compartmentalization of neurotransmitters in the brain and also exhibits a key propensity to get aggregated because of its internal constituents. The enhanced aggregation of the protein is then found to lead to elevated toxicity, which can result in neuron dysfunction in the patients. Another perspective or mechanism *via* which this protein tends to lead to PD is when the protein gets aggregated in the form of Lewy bodies inside the brain, it leads to the development of α-synucleinopathies (Kim et al., 2014). The molecular mechanism of cell–cell transmission is known to activate and push the accumulation of the protein, thus resulting in the reduction of DA production, causing an increase in the onset of the disorder (Mothes et al., 2010).

These mechanisms as described above have explained the causes behind the two most commonly observed neurodegenerative disorders. Further discussions thus evaluate and explain the recent advancements in the field of translational nanotheranostics for treating these disorders. The study presents the findings obtained from the approach used in different forms of modeling to deduce its applicability and limitations.

## Nanotheranostics and Its Development in the Context of Neurodegeneration

Nanotheranostics, as described by Zhang et al. (2018), includes the injection of the nanotheranostic agent *via* different drug particles, and after reaching the target area of the body, the shell of the medicine tends to disintegrate, resulting in the release of the agents. This helps in aiming at the molecules or neurons that are causing or leading to the disorder. According to Tripathy et al. (2018), the fact that the treatment is being given so much attention in the medical field is because it is an extremely aggressive treatment and targets directly the affected area in patients’ bodies. Moreover, it can be tailored according to the disease and needs of every individual, which leads to an enhancement in the overall practical applications of the approach, hence resulting in the provision of personalized treatment and medicine options (Assaraf et al., 2014). It has been argued that the therapy or treatment has become possible only because of the recent developments that have taken place in the field of chemistry and technologies as the chemical developments have led to the development of a phenomenon, wherein electromagnetic waves can be easily converted into processes that are medically relevant at the nanoscale because of the use of different metal nanoparticles (NPs) (Han et al., 2017). This chemical phenomenon is thus combined with the laser technologies that can help in penetration of the agents to a deeper level in tissues of human bodies, thus making the process simpler and much more effective. While these developments seem to make the application of nanotheranostic process extremely effective (Melancon et al., 2012), it is arguable whether the results that have been obtained until now are completely based on *in vitro* studies and the *in vivo* applicability still remains a challenge. The therapy has been tested in different situations and developed *via* animal, cell, and human modeling. Various tests have been conducted, and their outcomes are described below to explain the recent developments that have taken place in the field (McCarroll et al., 2014; Clift et al., 2015; Kievit et al., 2017; Zavaleta et al., 2018).

### Possible Methods to Overcome the Blood–Brain Barrier

There are a number of pharmacologically active substances that have the potential to treat CNS disorders, but due to the blood–brain barrier (BBB), they are not able to access their targets (Cipolla, 2009). Naturally, there are two transport methods (a) active and (b) passive by which molecules are absorbed by the BBB (Tsuji, 2005). Passive transport is a passive diffusion which follows two non-energetic transportation pathways known as paracellular and transcellular diffusion. Hydrophilic compounds are diffused between endothelial cells *via* paracellular diffusion, and small lipophilic molecules are diffused through endothelial cells *via* transcellular diffusion. Here, one thing is noted that lipid solubility plays a critical role in passive transport into the BBB, this method can be used for the chemical transformation of water-soluble molecules into lipid-soluble molecules that can cross the BBB. The drug molecules can be designed by the addition of lipid or functional moiety to them (Lu et al., 2014). Steroids and diphenhydramine are the examples of these types of drugs that can cross the BBB (Au-Yeung et al., 2006; De Gregori et al., 2012). Antibodies and erythropoietin are the examples of paracellular pathways that influence the molecules entering to some extent into the brain. These are usually the case of molecules that have long half-lives, small distribution volumes, and strong effects on the CNS (Banks, 2004). Passive diffusion of liposoluble molecules occurs *via* the transcellular transport (Abbott, 2005; Vos et al., 2015). The active transport requires energy for transport of therapeutic molecules to reach the CNS *via* crossing the BBB through biological gradients, i.e., as opioid analgesics, cardiac glycosides, and calcium channel blockers (Sanchez-Covarrubias et al., 2014). Passage of amino acids across the BBB is mediated by specialized transporters, i.e., glucose transporter (GLUT-1), that assist glucose to cross the brain (Du et al., 2014). Several amino acids, i.e., methionine, valine, histidine, and tyrosine, are required for crossing the BBB to gain access to the CNS *via* the transporter. L-DOPA or levodopa, an anti-PD drug, is the classical example of drug that enters the CNS by this mechanism which operates with large amino acid transporter 1 (LAT-1) (del Amo et al., 2008). Glucose, vitamins, and some peptides follow the same mechanism to cross the BBB to gain access to the CNS at a faster rate than expected (Banks, 2016). Transport through the BBB also occurs by efflux mechanisms. P-glycoprotein (Pgp) mechanism is the example efflux unwanted complexes, such as anticancer drugs and antibiotics (Begley, 2004; Huwyler et al., 2008). NP interactions with the blood coagulation can be engineered to carry anticoagulant drugs initiating factors to treat PD and AD. Coagulation system with nanomaterials and anticoagulant properties of NPs on the blood coagulation system represent significant concerns in the field of nanomedicine using physicochemical properties (e.g., size, charge, and hydrophobicity) that regulate their undesirable properties on the blood coagulation system to understand unwanted side effects that are used for industrial and medical applications because of their small size, toxicity, risk assessment, and management which warrant attention to clotting, reactions triggering inflammatory, immune responses, and hemolysis.

### Animal Modeling

Animal modeling has been one of the key approaches used by researchers to evaluate the impacts and implications of nanotheranostics, and the results of the same have been extremely positive. The modeling was started with small animals like mice and is now being scaled up to the human levels (Kievit and Zhang, 2011). One of the key carriers, whose impacta have been studied and evaluated on animals, is gold nanoparticles (AuNPs). AuNPs have been found to exhibit characteristics that make them extremely suitable for the purpose of nanotheranostics, and they can also be used in various shapes, thus providing higher flexibility to the clinicians (De Lau et al., 2006). The research carried out by Mieszawska et al. (2013) made use of the peptide-functionalized AuNPs to evaluate its impact on the gastric-releasing peptide (GRP) receptor. The AuNPs were conjugated along with an RGD-peptide and injected into small animals, i.e., mice, to evaluate the impacts of the elements on the animal. The application and injection of the NPs in the mice indicated higher tumor accumulation, thus providing a successful approach for treating tumors as well as NDs like AD (Ramanathan et al., 2018).

A similar study carried out by Muthu et al. (2014) included the testing on 24 mice, wherein AuNPs were injected in local tumors contained in those animals (Muthu et al., 2014). The mice were also divided into four different groups to be able to compare the results effectively; the groups contained agent-only group, laser-only group, control group, and lastly agent and laser group. The results of the study revealed that the use of gold coating on the medicine proved to be extremely beneficial in treating the animals and led to the destruction of tumor cells without having any impact on the animal (Muthu et al., 2014). Further explaining the fact that the practice or approach has been found to be effective for tumor ablation makes it effective for even treating diseases like PD wherein the proteins tend to accumulate and cause motor dysfunction, and thus the practice of nanotheranostics can be used to address those areas and destroy the over-accumulation of particles for better outcomes and results. Lee et al. (2015) have discussed that the developments that have taken place in nanotheranostics in South Korea and explained the way the application and modeling of the approach on animals like mice have given key insights into the benefits of the technique. One of the key nanotheranostic techniques that have been tested on mice is the synchrotron radiation x-ray computerized topography (SR-CT) approach, which is a high-speed imaging process (Lee et al., 2015). The approach was used on mice along with a colloidal hybrid silica NP, which resulted in an *in vivo* broad circulation and distribution of the same in the animal, thus leading to treatment of neuron-related defects. While these discussions reveal and confirm the effectiveness of nanotheranostics in treating neurodegenerative disorders in animals, Conde et al. (2015) argue that whether the same can be scaled up to humans or not is something that has not been verified yet.

### Cell Modeling

Another approach or practice that has been adopted in addition to the animal modeling approach is that of cell modeling, wherein different models are developed based on cell culture and characteristics of the human body to evaluate the impacts of the applied treatment. Researchers have made use of the cellular models of brains of different animals like mice to evaluate the impacts of nanotheranostics technique or approach on the factors causing the onset of neurodegenerative disorders (Chen et al., 2017; Kievit et al., 2017; Ulapane et al., 2017). Zhang et al. (2015) developed and made use of the cell-based models of different disorders like AD and PD and evaluated the impacts of NPs on macrophages on the cells contained in the model. The cell modeling based on the use of theranostics in microphage assisted in highlighting that the technique helps in ablation of macrophages, which tends to inhibit and reduce the overall inflammation in cells, thus resulting into treatment of the neurodegenerative disorder. However, the cell modeling-based results as presented by Zhao et al. (2017) highlight that the use of nanotheranostics has been found to be effective only at the preliminary stage on different cell structures of diseases and have not been very effective in the long run (Dilnawaz et al., 2012).

Cell modeling has been found to be majorly used for highlighting the benefits and application of the nanotheranostics technique in the diagnosis of neurodegenerative disorders. Mohan and Rapoport (2010) carried out multiple experiments, in which different nanotheranostic agents were developed and used for different diagnostic practices and approaches such as computed tomography and cellular imaging (Chowdhury et al., 2018). The results presented by Mohan and Rapoport (2010) highlighted that the inclusion of nanotheranostic agents and processes have led to the development of new practices, such as magnetic resonance spectroscopy, which help in better diagnosis of neurodegenerative disorders like AD and PD due to the enhanced ability of identifying the activation of CDK5 protein or identifying the areas of excessive accumulation of proteins causing PD. Niu et al. (2017) developed a silicon model of the cells of PD patients wherein the DA transporter cells were modeled. The use of nanotheranostic approach using NPs on the model was found to be aggressive and invasive and led to the stimulation of these transporter cells, thus resulting in the creation of higher DA which, as discussed earlier, has been found to be responsible for facilitating motor activities in humans.

These results obtained from human and cell modeling have given satisfactory results and outcomes but whether the approach can be used for clinical applications for treating neurodegenerative disorders is still doubtful. Recent developments have hence made use of a few human models to test the same, which are being described below.

### Human Modeling

García et al. (2017) carried out an evidence-based study of using nanotheranostics for personalized medicine in treating neurodegenerative disorders. The study made use of metallothioneins (MTs) as a drug discovery biomarker which was injected in patients suffering from neurodegenerative disorders. This nanotheranostic approach proved that the use of such biomarkers helps in targeting specific cells in human bodies, thus resulting in eradication of different malignancies and neural dysfunctions that usually results in disorders like AD and PD (García et al., 2017; Kumar et al., 2017; Kaushik et al., 2018b, 2019a,c; Pan et al., 2019). However, the study carried out by Ruozi et al. (2014) which focused on injecting AuNPs in voluntary AD patients revealed that the approach was effective and beneficial for early diagnosis of the neurodegenerative disorders, but was not yet completely able to treat the same.

Similar human modeling and test were carried out by Maysinger et al. (2015), and they studied the characteristics and problem areas of multiple human beings and identified different biomarkers on the basis of their conditions such that the unique characteristics of each of the NPs could be targeted at treating specific conditions. The study confirmed that while the use of these NPs is effective for diagnostics of neurodegenerative disorders, they still cannot be validated as an effective approach for treating them. Similar discussions as presented in a paper (Wang et al., 2017) confirm that the technique holds great promises for being used as a non-invasive technique for treating NDs like AD by targeting the specific problem areas and spots. Dual-functional NPs targeting amyloid plaques in the brains of AD mice have also been reported (Zhang et al., 2014; Kaushik et al., 2019b). However, these studies do not confirm the effectiveness of the method, and there are various limitations of the same that have been identified by experts. Some of the key limitations of the technique are hence being described in this review.

### Access of Nanoparticles to the Central Nervous System

**FIGURE 1:**
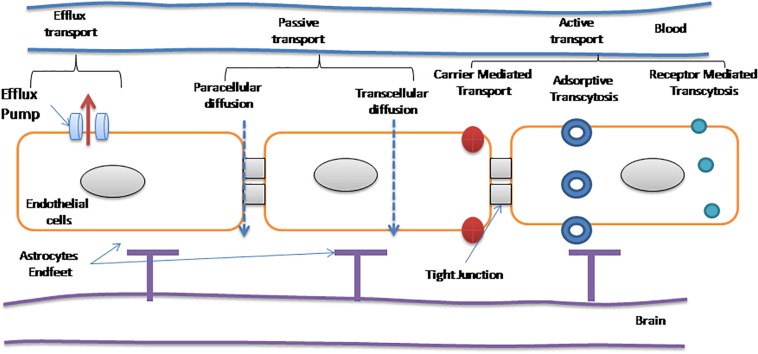
Different modes of transport across the blood–brain barrier (BBB; Teleanu et al., 2019).

With the advent of nanotechnology, new perspective to treat NDs has been opened for diagnostics as well as therapeutics purposes. Due to multifunctional and versatile structures of NPs, they can be utilized for brain drug delivery. But prior to their application, the major considerations are the surface chemistry, hydrophobicity, shape, size and charge, etc. An ideal NP should be biocompatible, has reduced toxicity, and has the ability to bind and transport drugs or therapeutics. It should not be degraded readily *in vivo* to control therapeutics release for prolonged time periods and can navigate *via* the BBB. All these features lead to those NPs which can penetrate the BBB with high efficacy (Goldsmith et al., 2014). Deliver NP conveniently and effectively has controlled to a deep examination of site-specific drug delivery, toward the convenient non-invasive delivery to specific sites of the delivered dose, to gain access to specific concentrate of therapeutic dose at the specific sites of pharmacological action. Currently, drugs/NPs gain access to the CNS *via* non-invasive methods, i.e., intranasal delivery that are based on drug modification to enhance the permeability of the BBB (Illum, 2003; Kaushik et al., 2018a) and invasive methods that impart direct intraventricular or intracerebral injection/implantation, infusion (Pardridge, 2016). Temporary disruption is also useful to some extent to cross the BBB (McDannold et al., 2012). The BBB disruption is broadly used to enhance drug delivery efficiency to the brain. For example, to treat certain CNS cancers, mannitol is used for osmotic opening to disrupt the BBB (KrolI and Neuwelt, 1998) and temporary pores are made open by using ultrasound in the BBB (Dasgupta et al., 2016). Recently, it has been evident that in the case of AD and multiple sclerosis, pathological BBB disruption occurs which leads to permeability for small therapeutic molecules (<1,000 Da) (Cheng et al., 2010). NPs have their intrinsic physical and chemical properties which decide the route of transport and mechanisms to cross the BBB (Chowdhury et al., 2018). When NPs are functionalized with an appropriate ligand, they can cross the BBB and are capable to distribute drugs in the diseased brain (Tosi et al., 2008; Kaushik and Wei, 2019). In Figure 1, several transport mechanisms of NPs have been illustrated and discussed. They can enhance the permeabilization of the BBB by opening tight junctions and letting drugs or drug-conjugated NPs infiltrate into the CNS (Choi et al., 2010); may follow transcytosis mechanism to pass through endothelial cells (Choi et al., 2010); and can be transported by endocytosis into endothelial cells (Kong et al., 2012). Neuropharmaceuticals research is the new emerging niche that is evolving gradually by understanding the receptor-mediated transcytosis and the adsorptive-mediated transcytosis mechanisms and by studying all the intrinsic physicochemical properties of NPs. This could lead to therapeutics that can cross the BBB more efficiently and can provide a patient-oriented therapy (Smith and Gumbleton, 2006).

## Nanoparticle Applications in Neurodegenerative Diseases

### Nanocomposite

Most of the AD treatments have focused on Aβ-targeted therapy. Chen et al. (2018) brought into picture that tau pathway is closely related to the development of AD (Kaushik et al., 2018b) (Uddin et al., 2018). They constructed a methylene blue-loaded multifunctional nanocomposite (CeNC/IONC/MSN-T807), which possessed a high binding affinity to hyper phosphorylated tau. The surface-decorated CeNC mitigated mitochondrial oxidative stress and suppressed tau hyper phosphorylation, whereas the loaded MB inhibited hyper phosphorylated tau aggregation. As a result, in comparison with the effectiveness of CeNC/IONC/MSN-T807 or MB alone, when both are combined to form a multifunctional nanocomposite, a synergistic therapeutic effect was achieved.

Siddique et al. (2016) studied the effect of alginate nanocomposite (BANC) on PD model flies. A significant decrease in lipid peroxidation and glutathione-S-transferase activity and an increase in glutathione content were observed in PD flies when exposed to BANC. Nevertheless, there was no gross morphological change in the brains of PD flies. The results suggested that BANC is effective in reducing the PD symptoms in these transgenic flies (Siddique et al., 2016).

Oxidative stress disorders have a wide influence on pulmonary, neurodegenerative, and autoimmune diseases and on many more disorders. Selenium (Se) is capable of suppressing oxidative stress disorder, but there is a very thin line between the toxicity and beneficial effects of Se, which makes it difficult and challenging to be used in therapy. Deng et al. (2017) developed porous Se:SiO_2_ nanocomposites which ensured controlled release of Se particles, having favorable biocompatibility and could effectively reduce reactive oxygen species (ROS) damage.

### Metal Nanoparticles

Nerve growth factor (NGF) is one of the most essential factors for neuronal growth. Nevertheless, its application in neurogeneration is limited due to slow diffusion and short half-life. Moreover, the main obstruction is that the NGF cannot be easily transported through the BBB because of its selective permeability of the BBB. Although there are many magnetic NPs that have been used as an effective nano-carrier for NGF, uncoated magnetic NPs face problems due to their instability in neuronal environment, aggregation, and cellular toxicity. To address these issues, Yuan et al. (2018) proposed that coating them with a thin and impenetrable shell with materials such as polymer, alloy, metals, etc. can solve the problem to some extent. NGF functionalized Au coated SPIO core NPs [Diameter (D): 20.8 nm] was synthesized by them. They selected gold (Au) stating it to be the best candidate due to its outstanding biocompatibility, high stabilities, and tunable surface function. This gold coating not only protects the iron oxide but also provides tunable surface functionalization with several ligands, such as proteins, aptamers, and peptides, due to which precise and controlled release of neuroregeneration can be achieved toward specific sites.

It has been reported that microRNA 141 (miR141) could silence the expression of lncRNA HOTAIR by binding to specific sites on lncRNA HOTAIR. Liu et al. (2018) mediated the high expression of microRNA141 in human amniotic epithelial stem cells by using super paramagnetic iron oxide NPs (SPIONs). They also reported that SPION-mediated overexpression of miR141 is able to promote the expression of brain-derived neurotrophic factor DAT and 5-TH are brain-derived neurotrophic factor (BDNF), DAT, and 5TH in human amniotic epithelial stem cells (HuAESCs)-derived dopaminergic neuron-like cells (iDNLCs). Their study confirmed that the efficiency of HuAESC differentiation into dopaminergic neuron-like cells could be highly improved by miR141 mediated by SPIONs.

Amyloid fibrils are closely associated with various NDs. The hindrance to the diagnosis and development of therapeutic strategies is the identification of fibrils at low concentrations (Kumar et al., 2018). Employed gold nanorods to detect the formation of amyloid fibrils based on α-synuclein. They observed no apparent interaction of gold nanorods with monomeric proteins. However, effective adsorption onto fibril structures *via* non-covalent interactions was reported. The strong dipolar coupling in helical Au nanorod promoted the intense chiral response as a result allowed them to detect amyloid fibrils as low as nanomolar concentrations.

Selective detection and quantification of DA is an important key to monitor NDs. However, the limit of detection for DA is in the lower nanometer range. It is significant to push the detection limit to few picomolar or lower. Cao et al. (2018) utilized DA DNA aptamer (DAAPT)–AuNP conjugate to enhance the surface plasmon resonance (SPR) signal (Xue et al., 2018). As a result, this enabled the detection and quantification of DA in the femtomolar to picomolar range. They reported that this is the lowest detection limit achieved for sensing of DA through SPR (Yuan et al., 2018).

### Quantum Dots

With emerging technology, it is indicated that the pathogenesis of PD is strongly related to the accumulation of α-synuclein aggregates. Anti-aggregation agent is yet to be achieved for this disease (Dong et al., 2009). Kim H. et al. (2018) showed that graphene quantum dots (GQDs) interact with α-synuclein to exhibit anti-amyloid activity. They reported that GQDs have notable potency not only in inhibiting fibrillization of α-synuclein, but they can also disaggregate mature fibrils with time. They also reported remarkable property of GQDs such as rescued neuronal death and synaptic loss. Moreover, GQDs were found out to help reduce Lewy body (LB)/Lewy neurite (LN) formation, ameliorate mitochondrial dysfunctions, and prevent neuron-to-neuron transmission of α-synuclein pathology induced by α-synuclein preformed fibrils (PFFs) in neurons. They proposed that GQD could function as an anti-aggregation agent, which provides a promising novel therapeutic target for the treatment of PD. The toxicity of QDs in the milieu and biological systems become a key point for the NP, and the potential toxicity of QDs on immunotoxicity of QDs is still unclear. Brain disorders’ major hurdle is BBB, many drugs are being proposed for brain disorders, which however fail because of their inability to cross the BBB. Hence, as the molecular structure of the BBB is better elucidated, several approaches are being evaluated, such as adsorptive-mediated transcytosis, inhibition of active efflux pumps, receptor-mediated transport, cell-mediated endocytosis, and delivery from microspheres, biodegradable wafers, and colloidal drug-carrier systems (e.g., liposomes, NPs, nanogels, dendrimers, micelles, nanoemulsions, polymersomes, exosomes, and QDs).

### Labeling and Biomarkers

Subtle alterations in synaptic connections and perturbed neuronal network functionality are the main characteristics to determine neurodevelopmental and neurodegenerative disorders. The presence of dendritic spines, micron-sized protrusions of the dendritic shaft that compartmentalize single synapses to fine-tune synaptic strength, is the hallmark of neuronal connectivity. However, it is hard to quantify spine density and morphology in mature neuronal networks due to the lack of targeted labeling strategies (Xiong et al., 2018) and optimize a method to deliver cell-impermeable compounds into selected cells based on spatially resolved nanoparticle-enhanced photoporation (SNAP) to resolve the above-stated problem (Xiong et al., 2018). They showed that efficient labeling of selected individual neurons and their spines in dense cultured networks can be achieved by SNAP without affecting short-term viability. SNAP holds promise for high-content screening campaigns of neuronal connectivity in the context of neurodevelopmental and neurodegenerative disorders.

### Biosensor

Multifactorial pathways affect AD, which is associated with the loss of nerve cells in the brain. This as a result leads to changes in related biomolecular levels as AD progresses. Therefore, for accuracy regarding AD, diagnosis is supposed to be done with combined detection of several lesions. Amyloid beta 1–40, 1–42, and τ (tau) protein can be used as main diagnostic target markers. Kim D. et al. (2018) reported highly selective biosensor for detection of AD core biomarkers using distinct localized SPR (LSPR) depending on AuNP shapes, which is called shape-code biosensor. This plasmonic sensor does not need any other method for separation of samples and identification of markers. It consists of only AuNPs and antibody. In addition to this, they reported that this is the first highly sensitive shape-code biosensor to detect AD biomarkers (Indrasekara et al., 2014).

## Limitations

One of the key limitations or challenges of making use of the nanotheranostic technique for treating neurodegenerative disorders, as identified by Indrasekara et al. (2014) is that every patient has a different genome and neural functioning and characteristics, and hence no single approach can be used to treat all of them. The practitioners not only are required to be able to diagnose the causes of these neurodegenerative disorders in every individual separately but also need to identify the suitable treatment for them, which is a challenging task and does not help in identifying one method or treatment technique that is suitable for all (De Lau et al., 2006).

Another challenge or limitation that has been identified by Kim et al. (2013) and suggests that the technique tends to become ineffective because the absorption time of the NPs used in the technique is very low and hence if the injection is not done adequately, these NPs might get absorbed in the blood or by other body parts instead of the area at where it was targeted. It has been found that the neurodegenerative disorders like AD and PD are caused by the neurodysfunction or accumulation of proteins and hence if the drug is not injected adequately, it will not be absorbed or used for treating the desired area (Kim et al., 2013). In fact, it has been argued that, in practice, only a small proportion or part of the drug gets transmitted and transferred to the affected areas and hence the treatment has not yet been established to be completely effective (Rahman et al., 2015).

Another limitation as identified is that the treatment cannot be monitored effectively and efficiently, which imposes a big challenge for the practitioners because they are not able to monitor the progress and impacts of the treatment that they are giving to the patients. This thus makes the treatment method and technique ineffective. The treatment is also extremely expensive currently. Diou et al. (2012) discuss that most of the research done in the field has failed massively, which has led to an increase in the overall costs of research, thus also leading to higher costs of delivery of the same in the market. These limitations are thus needed to be overcome in order to enhance the applicability of the technique (Hans and Lowman, 2002; Diou et al., 2012; Ilinskaya and Dobrovolskaia, 2013; Luo et al., 2015; Liu et al., 2017; Radhakrishnan and Kamalasanan, 2018; Khan et al., 2019).

## Conclusion

The molecular changes and reactions have been found to be the key causes of NDs. The research in this field predominantly focuses on genetic mutations, building up of toxic proteins, and formation of neurotoxic molecules. The discussions presented in this study reveal that the nanotheranostics approach has a huge potential in the field of medicine for treating different neurodegenerative disorders. Animal, cell, and human modeling using nanotheranostics approaches for the treatment of neurodegenerative disorders has revolutionized the field. Nanocomposite, metal NPs, and QDs are well reported to treat these neurodegenerative disorders. Limitations that are associated with these NPs are needed to be overcome. Although the studies that have been carried out for the field until now have not provided any significant results to be scaled up to humans, their impacts on the reduction of molecular activities causing neurodegenerative disorders have been significant and hence the approach has a strong potential for developing significant outcomes in the long run.

## Author Contributions

All authors conceptualized the ideas, conducted the literature search, prepared the figure, drafted the manuscript, and read and approved the final manuscript.

## Conflict of Interest

The authors declare that the research was conducted in the absence of any commercial or financial relationships that could be construed as a potential conflict of interest.
